# Supplementary material for: Development of INER-PP-F11N as the Peptide-Radionuclide Conjugate Drug Against CCK2 Receptor-Overexpressing Tumors
Source: Int J Mol Sci. 2025 Jul 8;26(14):6565. doi: 10.3390/ijms26146565 (PMC12294753; doi:10.3390/ijms26146565)
Supplement: Supplementary file 1 [file ijms-26-06565-s001.zip › ijms-3704864-supplementary.docx]

**Figure S1. Certificate of Analysis (CoA) of radiolabeled peptide precursors at the time of purchase.** (A) CoA of DOTA-PP-F11N showing >99.3% purity with retention time (RT) of 5.1 min by HPLC. (B) CoA of DOTA-INER-PP-F11N-1 showing 98.4% purity. (C) CoA of DOTA-INER-PP-F11N-2 showing 98.7% radiochemical purity. These data confirm the physical-chemical integrity of each compound upon procurement, in response to reviewer comments regarding the original quality of the materials used.

**Figure S2. Radiochemical stability of [Lu-177]DOTA-INER-PP-F11N in formulation buffer at different time points.** Radio-HPLC analysis was performed at 0, 4, and 144 hours post-synthesis. The radiochemical purity remained above 96% at all time points except Lu-177 DOTA-PP-F11N, indicating high formulation stability of the radiolabeled peptide under storage conditions. These results support the suitability of the drug formulation for extended experimental use.

**Figure S3. Correlation between CCK2R expression and overall survival in patients with various cancer types by public open database.** (A) High CCK2R expression correlated with poor survival outcomes in glioma patients (n=153). The 3 years-survival rate was 0% and 12% for high and low CCK2R expression group, respectively (P=0.0023). (B) 5-years survival of endometrium cancer patients (n=541). Compared to low expression group, patients with high CCK2R expression had a significantly shorter survival (69% vs 73%, P=0.046). (C) 5-years survival of pancreatic cancer patients (n=176). Compared to low expression group, patients with high CCK2R expression had a significantly longer survival (33% vs 15%, P=0.03). (D) The 5-years survival rate of ovarian cancer patients (n=373) with high expression and low expression of CCK2R was not significantly different (39% vs 29%, P=0.08). (E) The 5-years survival rate of lung cancer patients (n=994) with high expression and low expression of CCK2R was not significantly different (41% vs 49%, P=0.19). (F) The 5-years survival rate of breast cancer patients (n=1,075) with high expression and low expression of CCK2R was not significantly different (84% vs 81%, P=0.28) (all by log-rank test).

*Public open database: The CCK2R expression and clinical parameters of various cancer types in TCGA and GTEx were obtained from the UCSC Xena database (https://xenabrowser.net/datapages/). We obtained clinicopathological information from the database, including tumor grade, histology, stages at diagnosis, treatments administered, recurrence, and survival outcomes. The period from cancer diagnosis to death or last visit was defined as OS. The relationship between CCK2R expression and the prognosis of various cancer patients was analyzed using the Kaplan-Meier plotter (https://kmplot.com/analysis/).
