# Supplementary figures and images for: Development of INER-PP-F11N as the Peptide-Radionuclide Conjugate Drug Against CCK2 Receptor-Overexpressing Tumors
Source: Int J Mol Sci. 2025 Jul 8;26(14):6565. doi: 10.3390/ijms26146565 (PMC12294753; doi:10.3390/ijms26146565)

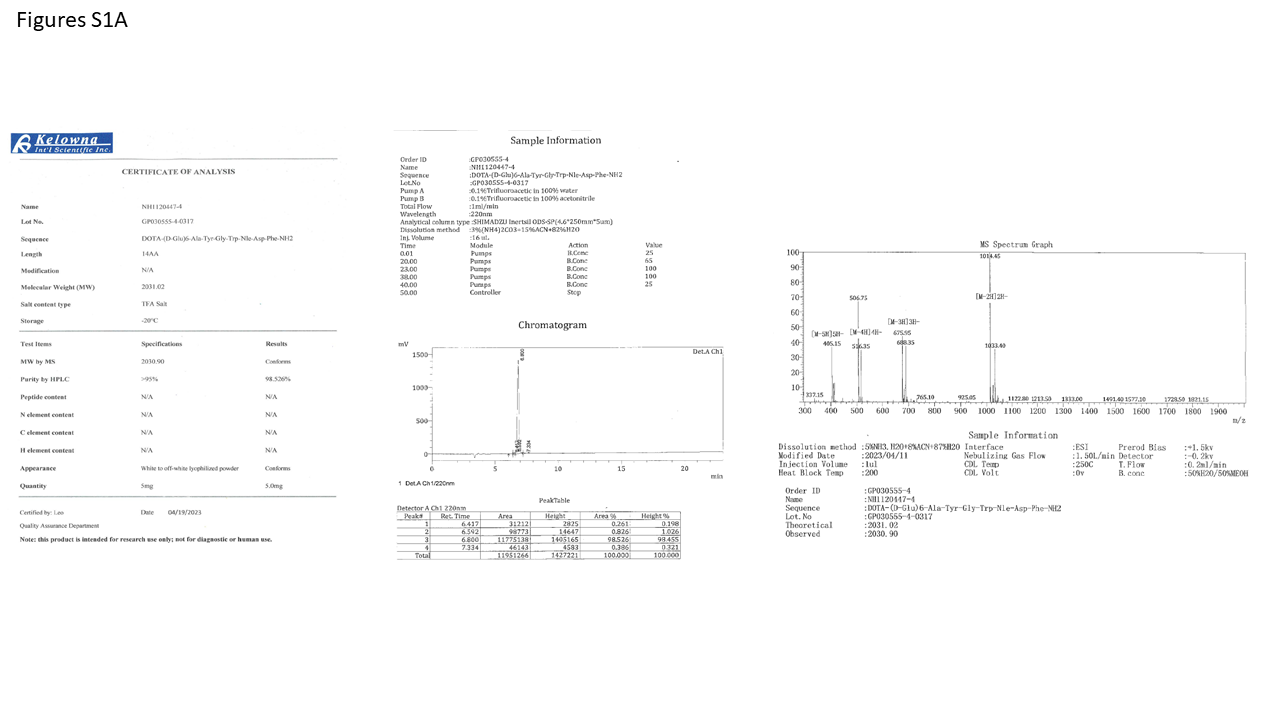

Supplement: Supplementary file 1 [file ijms-26-06565-s001.zip › Figure S1A 20250624.tif]

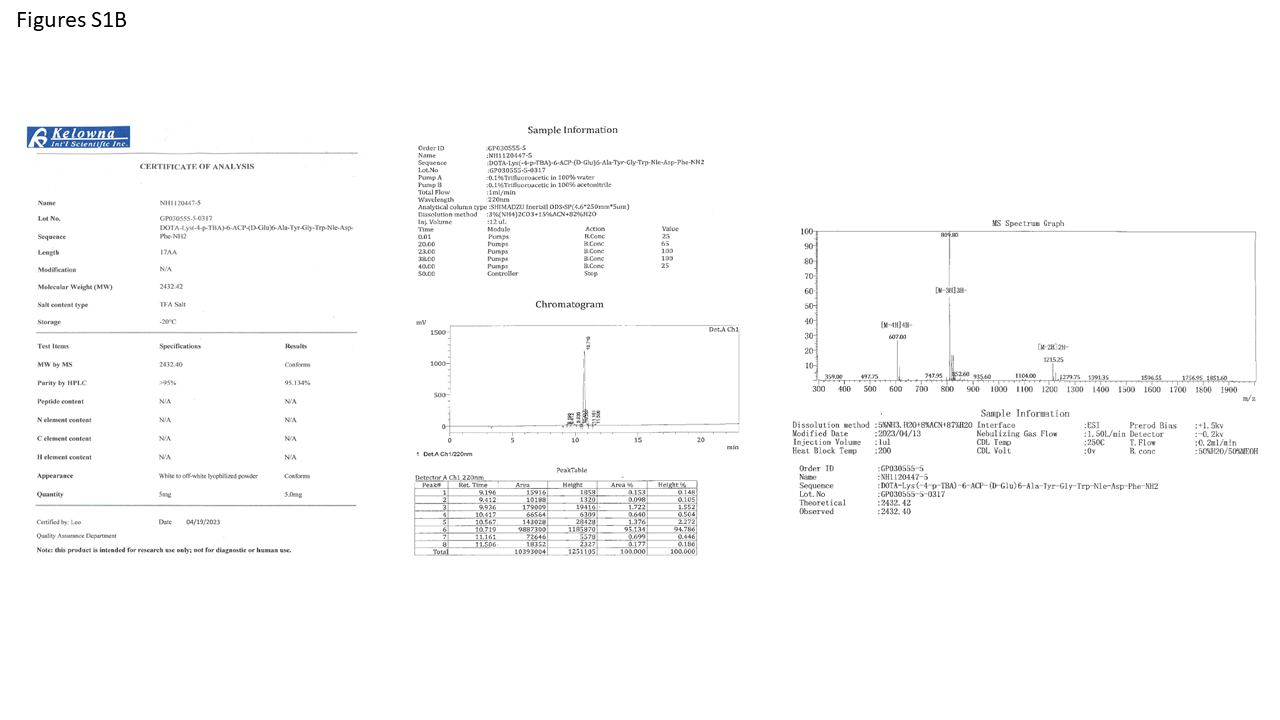

Supplement: Supplementary file 1 [file ijms-26-06565-s001.zip › Figure S1B 20250624.tif]

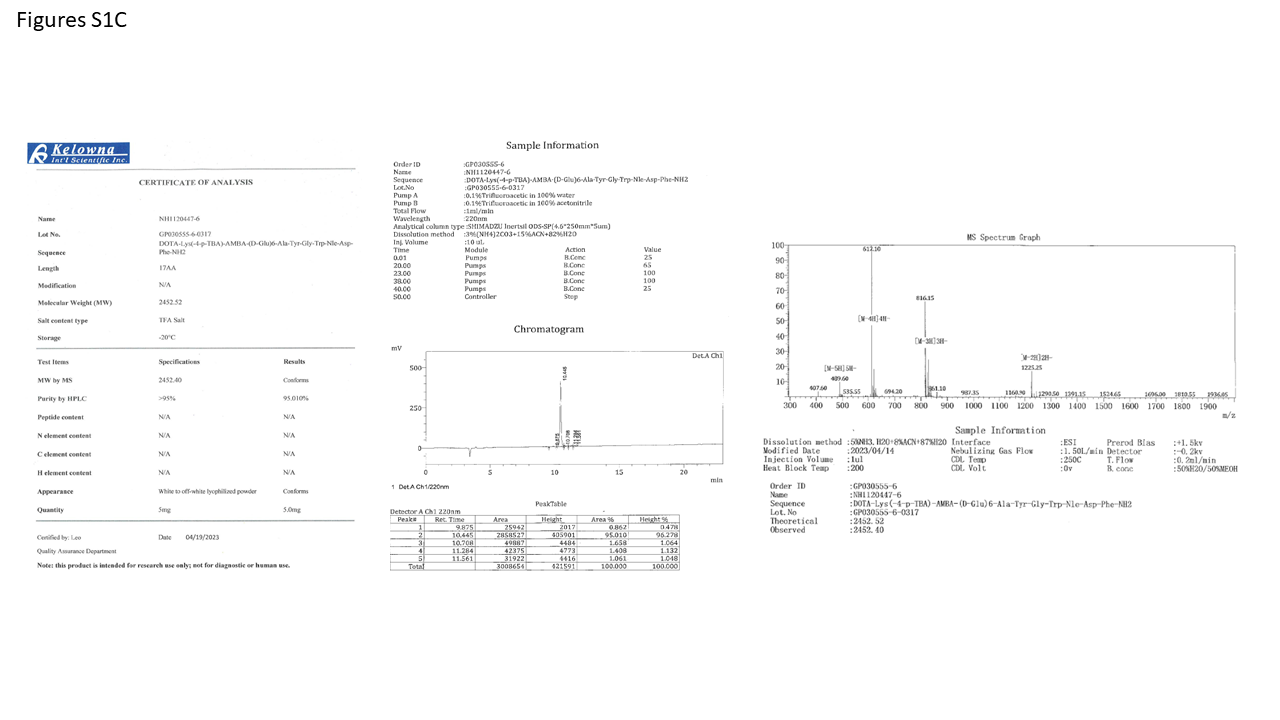

Supplement: Supplementary file 1 [file ijms-26-06565-s001.zip › Figure S1C 20250624.tif]

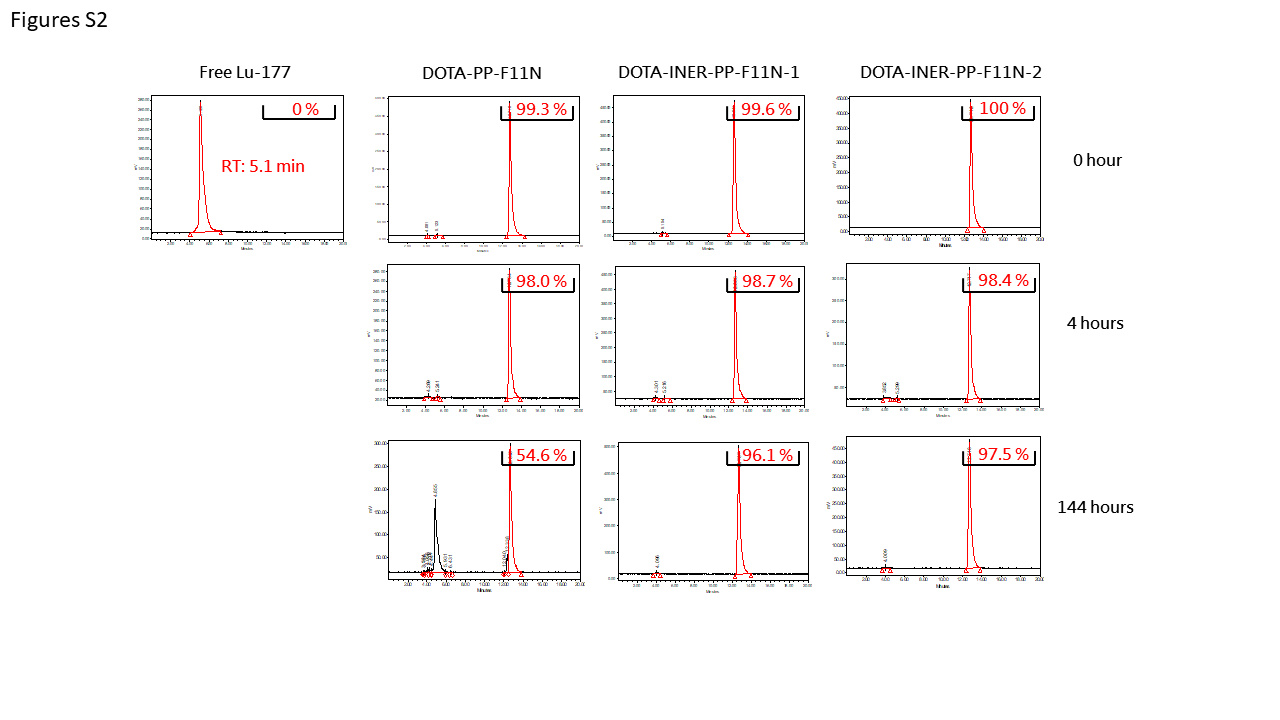

Supplement: Supplementary file 1 [file ijms-26-06565-s001.zip › Figure S2 20250624.tif]

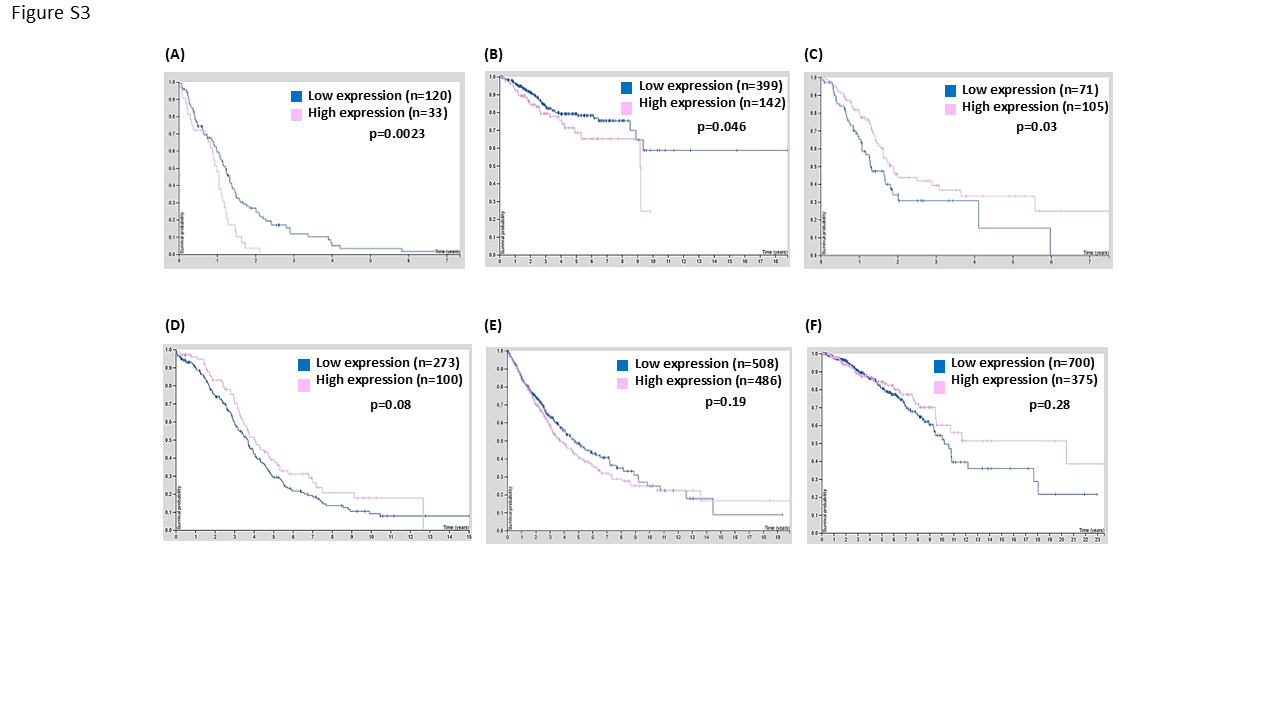

Supplement: Supplementary file 1 [file ijms-26-06565-s001.zip › Figure S3 20250624.tif]
